# Angina, “Normal” Coronary Angiography, and Vascular Dysfunction: Risk Assessment Strategies

**DOI:** 10.1371/journal.pmed.0040012

**Published:** 2007-02-27

**Authors:** Raffaele Bugiardini, Lina Badimon, Peter Collins, Raimund Erbel, Kim Fox, Christian Hamm, Fausto Pinto, Annika Rosengren, Christodoulos Stefanadis, Lars Wallentin, Frans Van de Werf

## Abstract

The authors discuss how to stratify risk in patients with chest pain and a normal coronary angiogram.

Chest pain may be associated with coronary arteries that appear “normal.” Normal is defined here as no visible disease or luminal irregularities (less than 50%) as judged visually at coronary angiography. Normal angiography in patients with chest pain is five times more common in women than in men [1]. Among patients with chest pain and normal angiography, an unknown number are suffering from cardiac pain of ischemic origin. Uncertainty is often difficult to allay, for medical attendants as well as for patients, resulting in perpetuation of symptoms, difficulties in management, and establishment of risk of subsequent coronary events [2]. In this article, we discuss how to stratify risk in patients with chest pain and a normal coronary angiogram. We based our article on a literature review, using the key words “angina with normal angiography,” “angina with normal coronary arteries,” “non-obstructive coronary disease,” or “chest pain of non-cardiac origin,” plus “[a]etiology,” “pathophysiology,” “diagnosis,” “classification,” “prognosis,” or “therapy.” A longer, more detailed version of this paper is found in the supplementary file S1.

## Definition

Over the past ten years, many terms have been proposed to label patients suffering chest pain due to myocardial ischemia despite normal coronary angiography, including syndrome X [3], microvascular angina [4], and non-atherosclerotic myocardial ischemia [5]. The term syndrome X refers only to patients showing ST-segment depression on an exercise electrocardiogram (ECG) with completely smooth coronary arteries at angiography. More recently it has come to be used also for the metabolic syndrome, comprising hypertension, insulin resistance, elevated triglycerides, low levels of high-density-lipoproteins, and obesity. The term microvascular angina may have outlived its usefulness, because patients with chest pain and normal angiography often exhibit a disorder not only of the coronary microcirculation, but also of the entire coronary artery and peripheral circulation [6,7].

**Figure 1:**
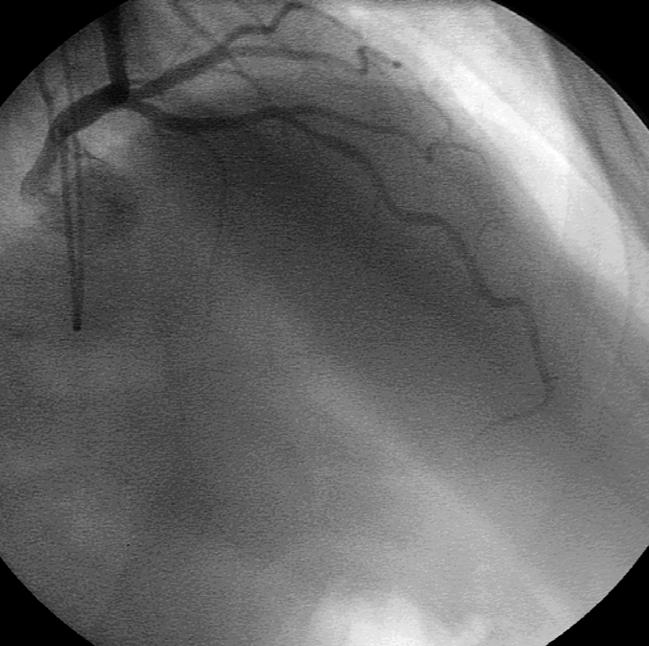
Normal Coronary Angiogram in a Patient with Chest Pain

## Mechanisms

The idea that some forms of ischemic heart disease may be caused by abnormalities of the microcirculatory vessels is not new. This idea was proposed 16 years ago as a cause of angina pectoris [4]. Although the importance of microcirculation for the regulation of coronary blood flow has become clearer, pathophysiological explanations of the disease process are still poorly understood.

### Vascular dysfunction.

Abnormality of both endothelium-dependent and independent vasodilatation due to early atheroma may be a cause of vascular dysfunction [7–11].

A normally functioning vascular endothelium is required for appropriate dilatation of arteries during exercise [12]. Endothelial dysfunction could underlie a nonspecific enhancement of the response to all vasoconstrictor stimuli [9]. Impairment of endothelium-dependent dilatation shifts a net dilator response to sympathetic stimulation to a net constrictor response [10].

### “Occult” atherosclerosis.

Recent pathophysiological studies show that the current concept of myocardial ischemia induced by epicardial coronary functional or fixed luminal narrowing should be renewed. Acute coronary syndromes often result from disruption of modestly stenotic plaques, not detectable by angiography, but only by intravascular ultrasound [13–15]. Plaque rupture and erosion often leads to thrombotic complications [14], or occasionally plaques may rupture and debris may be washed downstream, leading to peripheral coronary microembolization often associated with rhythm abnormalities [16]. Disturbed microvascular integrity could therefore be due to “hidden” epicardial atherosclerosis and its complications.

## Prognosis

The prognosis of patients with chest pain and normal or near normal coronary arteries at angiography is not as benign as reported by preliminary cohort studies. Many such patients are at an increased risk of myocardial infarction and cardiac death. In fact, 2% of patients—men and women—with unstable angina and non-ST-segment elevation (and normal or near normal coronary arteries at angiography) die within one year of their admission [17].

The risk is not invariably high and the Thrombolysis in Myocardial Infarction (TIMI) Risk Score helps to estimate risk. The rate of death or non-fatal myocardial infarction climbs from 0.6% in patients with a TIMI score of 1, to 4.1% in those with a score of 4 or more. Adverse outcomes may also occur in less acute clinical conditions. The combined risk of death, myocardial infarction, stroke, and heart failure is over 2% per year in women with a history of chronic chest pain symptoms persisting more than one year [18]. The population is heterogeneous and the presence of coronary vascular dysfunction may predict the likelihood of patients developing coronary events.

## Coronary Vascular Dysfunction

Coronary vascular dysfunction due to abnormal endothelium-dependent coronary vasodilatation is predictive of adverse outcomes [7,8,19–21]. Conversely, impaired endothelial-independent vasodilatation predicts favorable outcomes [19]. The reason for this discrepancy is not known. One might hypothesize that a blunted response to exogenous nitric oxide donors (coronary endothelial-independent vasodilatation) might simply reflect the presence of atherosclerosis, leading to increased stiffness of the vessel wall [20]. Conversely, endothelial dysfunction and atherosclerosis, although causally related, are distinct problems and may exist separately. Coronary endothelial vasoreactivity may indeed represent an index that combines information about the underlying atherosclerotic process and the overall stress imposed by risk factors on the arterial wall.

### Endothelial function.

Coronary endothelial dysfunction in patients with significant coronary artery disease (CAD) provides prognostic value independent of the traditional cardiovascular risk factor assessment [22]. Recent investigations addressed this issue in patients with chest pain and normal coronary angiography, and showed that 30% of patients with chest pain and severe endothelial dysfunction, as assessed by intracoronary acetylcholine testing, developed angiographically visible atherosclerosis during a ten-year follow-up [8].

Other studies evaluated patients with normal or near normal coronary angiography for a mean follow-up of four to six years [7,19–21]. Endpoints of analysis were cardiovascular death, acute myocardial infarction, unstable angina pectoris, and acute ischemic stroke. Acute vascular events occurred in 4.5% of patients. When patients were divided into two groups with either normal or abnormal endothelial function, the event rate of patients with endothelial dysfunction increased up to 14%.

## Clinical Decision Making

Nearly 4 million cardiac catheterizations are performed annually in United States hospitals alone, with one in ten discharges having undergone coronary arteriography [23]. More than 20% of patients aged 45–79 years admitted to hospital receive diagnostic cardiac catheterization [23]. More than 50% of women who are referred for cardiac catheterization because of chest pain show non-obstructive coronary lesions or smooth coronary arteries. [24]. Most of these patients have no diagnosis of angina at the time of discharge.

Epidemiological studies show that undiagnosed angina is costly in terms of mortality, morbidity, and health-care utilization [25]. In one study, over 65% of patients were re-hospitalized for chest pain during an 11-year follow-up and still remained without a diagnosis of angina [25]. Among those with an abnormal ECG result, the absolute risk of non-fatal myocardial infarction was similar in those with and without a diagnosis of angina (16% versus 15%). Also, compared with apparently healthy people, those with undiagnosed and diagnosed angina had a 2.4 and 3.2 times greater risk, respectively, of impaired physical functioning [25]. These findings underscore the importance of greater vigilance in identifying the causes of angina in patients with normal angiography

### Symptoms and quality of life.

Chest pain is the most common symptom of coronary atherosclerosis prompting patients to seek medical attention. Patients with normal or near normal coronary arteries may present with stable or unstable symptoms [8,17,18,26]. At one extreme are patients in whom angina develops every time the workload of the heart is increased beyond a fairly fixed threshold; at the other extreme are patients who are not necessarily restricted in their physical activity by angina, but suffer rest pain without an obvious cause. Both women and men most commonly report typical angina [8,17]. Along with severe and often unpredictable symptoms, chest pain is often associated with increased psychological morbidity, debilitating symptoms, and a poor quality of life [27–29]

**In clinical practice, symptoms in most of these patients are often indistinguishable from those with obstructive CAD. More information and additional testing is needed. It is always important to establish whether or not myocardial ischemia is present.**

### Noninvasive testing.

Use of imaging rather than non-imaging stress testing may be helpful in the identification of microvascular flow obstruction. Magnetic resonance imaging and gated-single photon emission computed tomography (SPECT) show a substantial overlap in detection of adequate or inadequate flow reserve patterns [30]. In patients with a normal resting ECG, exercise stress myocardial perfusion SPECT yields additional prognostic value over clinical, historical, and exercise treadmill test data for the prediction of coronary events [31]. SPECT abnormality is predictive of both cardiac death and myocardial infarction in patients with obstructive and non-obstructive CAD [32]. Perfusion imaging, unlike angiography, closely correlates with flow reserve and reflects function of the epicardial conduit arteries as well as normal capacity of the resistance vessels [31].

A growing number of centers offer sophisticated cardiac diagnostic tests to aid diagnosis and management of patients with ischemic heart disease. These tests include magnetic resonance imaging, high frequency transthoracic Doppler harmonic echocardiography, and transesophageal echocardiography with Doppler imaging. [30,33,34]

**In clinical practice, ischemia with standard SPECT testing identifies symptomatic patients at relatively high risk of a subsequent cardiac event.**

### Routine coronary angiography.

Data analysis of patients undergoing their first coronary angiography over a three-year period showed that 32% had entirely normal coronary angiograms and an additional 15% had less than a 50% stenosis in any of the major vessels [35]. The frequency of non-obstructive CAD is higher in black patients and in women. Chronic stable angina is the most common clinical presentation in patients without obstructive CAD. Many of these patients do not have angiographically visible plaques, but they have coronary disease.

**In clinical practice, new effective technologies may provide better understanding of the mechanisms that account for chest pain in patients with normal or near normal angiography.**

### New invasive technologies.

Sensor-tipped guidewires have enabled cardiologists to identify physiological measures such as absolute coronary flow reserve, relative coronary flow reserve, and pressure-derived fractional flow reserve. The most frequently used measure is absolute coronary flow reserve. Intravascular ultrasound (IVUS) is a relatively new modality for assessment of atherosclerotic disease burden. IVUS showed that atherosclerotic disease is diffuse and involves the entire arterial tree, including multiple plaques that are not associated with vessel narrowing.

**Early signs of atherosclerosis can be detected by IVUS. This may have important therapeutic and prognostic implications [36].**

### Endothelial function testing.

Over the past several years, sophisticated techniques have evolved to measure endothelium-dependent vasodilatation in the brachial and coronary arteries [37]. Peripheral endothelial function testing through measurements of flow-mediated vasodilatation is attractive because it is non-invasive and allows repeated measurements. Coronary artery endothelial function is most commonly assessed by intracoronary infusion of acetylcholine, which, acting via muscarinic receptors on endothelial cells, causes release of nitric oxide and coronary artery dilation [6,7,11,12]. Reduced vasodilatory response of the coronary microcirculation or paradoxical vasoconstriction of the epicardial vessels is a sign of coronary endothelial dysfunction. The relationship between peripheral and coronary endothelial function is still under scrutiny.

**Intracoronary acetylcholine testing may be used for patients who, during angiography, turn out not to have obstructed coronary vessels. Endothelial function measurement by peripheral testing may be a useful surrogate to reduce costs, inconvenience, and risks associated with invasive techniques during medical follow-up.**

## Approach to Management of Individual Patients

There are currently no critical pathways specifically developed for patients with chest pain and normal angiograms. So far, physicians apply (in varying proportions) pathophysiologic reasoning, personal clinical experience, and published research in the development of their own clinical approaches. Treatment objectives include symptom relief and risk factor identification and management with the objective of reducing progression in the underlying disease process with enhancement of event-free survival.

### Symptom relief.

At present, all patients with chronic, stable ischemic heart disease should receive long-term treatment with beta-blockers to control angina even if they have normal or near normal coronary arteries at angiography [38]. Assuming that the patient has no evidence of myocardial ischemia and/or vascular dysfunction on image stress testing, they can be discharged on imipramine in an attempt to manage chest pain. Patients without evidence of a cardiac etiology may require referral for evaluation of noncardiac causes of chest pain.

### Risk factor control and enhancement of event-free survival.

Clinical guidelines recommend managing all patients with acute coronary syndromes with a standard set of therapies, independent of what coronary anatomy looks like [39]. Chronic stable symptoms may require a more selective intervention. Therapy with statins, and perhaps angiotensin-converting enzyme inhibitors, should be used for patients with evidence of coronary endothelial dysfunction and/or atherosclerotic plaques within the arterial wall detected by IVUS [38,40].

A negative angiographic test cannot alleviate the call for intensive lifestyle changes in these patients. The medical community should become increasingly aware that atherosclerosis poses a serious health risk even in its mild form. It is important to understand that the presence of coronary endothelial dysfunction and/or atherosclerotic plaques within the arterial wall is not normal and deserves medical intervention.

## A Call for Clinical Trials

We recommend the development of large-scale prospective collaborative clinical trials as well as retrospective analyses of already completed trials, in which the results of angiography may be available in at least a portion of patients. Large numbers of patients will need to be studied, since relatively low-risk patients will be included. These studies require considerable communication among all parties: researchers, physicians, hospital administrators, patient groups, and policy planners. This communication can only be realized by close collaboration between important scientific institutions playing a prominent role in research funding and program development. A call to action for risk assessment strategies in this area is warranted.

## Supplementary Information

Text S1 Longer, more detailed version of article (119 KB DOC).

## Abbreviations

CAD: coronary artery disease
ECG: electrocardiogram
IVUS: intravascular ultrasound
SPECT: single photon emission computed tomography
TIMI: Thrombolysis in Myocardial Infarction
