# Supplementary material for: Angina, “Normal” Coronary Angiography, and Vascular Dysfunction: Risk Assessment Strategies
Source: PLoS Med. 2007 Feb 27;4(2):e12. doi: 10.1371/journal.pmed.0040012 (PMC1808079; doi:10.1371/journal.pmed.0040012)
Supplement: Text S1 — (119 KB DOC). [file pmed.0040012.sd001.doc]

### Review

### Angina, “Normal” Coronary Angiography and Vascular Dysfunction: Risk Assessment Strategies.

**Authors:** Raffaele Bugiardini (1), Lina Badimon (2), Peter Collins (3), Raimund Erbel (4), Kim Fox (3), Christian Hamm (5), Fausto Pinto (6), Annika Rosengren (7), Christodoulos Stefanadis (8), Lars Wallentin (9), Frans Van de Werf (10).

**Author Affiliations:** (1). Department of Internal Medicine, Cardio-Angiology and Hepatology, University of Bologna, Italy. (2) Cardiovascular Research Center, CSIC-ICCC, Hospital de la Santa Creu i Sant Pau, Barcelona, Spain. (3) Department of Cardiac Medicine, National Heart and Lung Institute and Royal Brompton Hospital, Imperial College London, London, UK. (4) Department of Cardiology, University Clinic Essen, Germany. (5) Department of Cardiology, Kerckhoff Heart Center, Bad Nauheim, Germany. (6) Department Cardiology-University Hospital Sta Maria, Lisboa-Portugal. (7) Department of Medicine Sahlgrenska University Hospital/Ostra Goteborg, Sweden. (8) Athens University Medical School, Hippokration Hospital, Cardiology Department, Athens, Greece. (9) Uppsala Clinical Research Center, University Hospital, Uppsala, Sweden. (10) University of Leuven, Leuven, Belgium.

**Corresponding Author:**

Raffaele Bugiardini, Department of Internal Medicine, Cardio-Angiology and Hepatology, Padiglione 11, University of Bologna, Via Massarenti 9, Bologna 40138, Italy.

Phone and fax: +39051347290 E-mail: raffaele.bugiardini@unibo.it

**Word count (for text only):** 4771

# Abstract

The syndrome of chest pain with normal or near normal coronary angiography is common and is a troublesome management problem for physicians. Patients often complain of chest pain and dis­ability for years, and the morbidity is considerable. Adverse events may include cardiac death, nonfatal myocardial infarction, and revascularization. A more adverse prognosis applies to patients having vascular endothelial dysfunction. A call to action for risk assessment strategies in this area is warranted.

## Article Outline

Definition

Pathophysiology

Reduced Coronary Flow Reserve and Myocardial Ischemia

Vascular Dysfunction

Autonomic Nervous System Activity

Hidden Atherosclerosis

Prognosis

Coronary Endothelial Dysfunction

Clinical Decision Making

Symptoms and Quality of Life

Noninvasive Testing

Routine Coronary Angiography

Coronary Flow and Pressure Measurements

Intracoronary Ultrasound

Markers of Early Stage Atherosclerosis and Disease Activity

Endothelial Function Testing

Approach to Management of Individual Patients

Symptom Relief

Risk Factor Control and Enhancement of Event-free Survival

Nonobstructive Coronary Disease in Women

Perspectives

Search Strategy and Selection Criteria

References

# Introduction

Chest pain may be associated with “*normal*” appearing coronary arteries. “*Normal*” is defined as no visible disease or lumen irregularities (<50%) as judged visually at coronary angiography. This finding is five times more common in women than in men (1). Among patients with chest pain and normal angiography an unknown number are suffering from cardiac pain of ischemic origin. Uncertainty is often difficult to allay, for medical attendants as well as for patients, resulting in perpetuation of symptoms, difficulties in management and establishment of risk of subsequent coronary events (2,3).

There is a need for improvement of the diagnosis of chest pain with an ischemic etiology and for evaluation of its impact on clinical outcome.

### Definition

Over the past 10 years, many terms have been proposed to label patients suffering chest pain due to myocardial ischemia, including syndrome X (4), microvascular angina (5), and non-atherosclerotic myocardial ischemia (6).

The term syndrome X refers only to patients showing ST-segment depression on an exercise ECG with completely smooth coronary arteries at angiography. More recently it has come to be used also for the metabolic syndrome, comprising hypertension, insulin resistance, elevated triglycerides, low high-density-lipoprotein and obesity (7). The term microvascular angina may have outlived its usefulness, because these patients often exhibit a disorder not only of the coronary microcirculation, but also of the entire coronary artery and peripheral circulation (8,9).

Increasing evidence suggests that many patients with angina pectoris and normal or near normal angiography already have dysfunctional arteries and early stage coronary atherosclerosis (10-12). The term non-atherosclerotic myocardial ischemia should, therefore, be ruled out.

The authors propose the term “angina with coronary vascular dysfunction” as the most appropriate description of this syndrome.

# Mechanisms

The idea that some forms of ischemic heart disease may be caused by abnormalities of the microcirculatory vessels is not new. It was proposed 16 years ago as a cause of angina pectoris (5). Although the importance of microcirculation for the regulation of coronary blood flow has become clearer (10), pathophysiological explanations of the disease process are still poorly understood.

**Reduced Coronary Flow Reserve and Myocardial Ischemia**

Coronary flow reserve can be defined as the amount of coronary blood flow increase in response to maximal arteriolar dilation induced by metabolic or pharmacological stimuli. A reduced coronary flow reserve can be seen in 20 to 50% of patients with chest pain and normal angiography (5,8,14-17).

Early studies suggested that chest pain could be non-ischemic in origin (18), even in the presence of a reduced coronary flow reserve (14). This view was supported by data showing no increase in myocardial lactate produc­tion (14) or decrease in coronary sinus oxygen saturation (19) during atrial pacing, and no abnormalities of left ventricular wall motion during chest pain precipitated by dobutamine or dipyridamole testing (20,21).

On the basis of these observations, some authors postulated that angina with normal angiography could be characterized by the paradox of a reduced coronary flow reserve in the absence of "true" myocardial ischemia (22). This hypothesis can hardly be conceived in the classic pathophysiological context of ischemic heart disease. Recent investigations have shed light on this issue by using new sensitive markers of hypoxia, including trans-­myocardial lipoperoxide production (23) and abnormalities in myocardial phosphorus metabolism (24). These studies demonstrated that coronary microvascular dysfunction might be severe enough to induce myocardial ischemia in at least 20% of patients with chest pain and normal or near normal angiography (24).

## Vascular Dysfunction

Abnormality of both endothelium-dependent and -independent vasodilatation due to early atheroma may be a cause of vascular dysfunction (9,10,15,25-28). A possible unifying hypothesis explaining the relationship between vascular dysfunction and myocardial ischemia is: *“submaximal increase in myocardial blood flow during effort could often be inadequate to match changes in oxygen demand and, therefore, may be a cause of angina”.* Attempts to develop such a unifying hypothesis, however, are at variance with the clinical picture. Many patients experience angina at rest, which implies a primary reduction of coronary blood flow and oxygen supply. How an impairment of vasodilatation can result in decreased myocardial perfusion at rest is still unknown. Mechanisms are largely speculative but may be summarized as follows.

A normally functioning vascular endothelium is required for appropriate dilatation of arteries during exercise (29). Endothelial dysfunction could underlie a nonspecific enhancement of the response to all vasoconstrictor stimuli. Impairment of endothelium-­dependent dilatation shifts a net dilator response to sympathetic stimulation to a net constrictor response (30-32).

Inappropriate vascular function accompanying heightened sympathetic activity may lead to epicardial and/or microvascular constriction with primary reduction of coronary blood flow, which may explain episodes of chest pain at rest.

## Autonomic Nervous System Activity

Activation of the sympathetic nervous system has been implicated as a trigger for microvascular constriction. Analysis of electrocardiographic monitoring data has revealed that many angina episodes during daily life occur in association with mental arousal, rather than with physical exercise (33). Myocardial ischemia occurs predominantly during waking hours, none during sleep; 25% during effort; 35% during minimal physical activity; 28% during activities demanding routine mental work such as conversation, reading, or watching television; and 10% during activities not well specified. An increase in heart rate (averaging 20 to 30 beats per minute) accompanies 95% of all ischemic episodes. However, on other days or at different hours of the same day, ischemia develops at a heart rate that otherwise is well tolerated (34). Concurrent changes in blood pressure, ventricular volume, and wall tension may modify the relationship between heart rate and myocardial oxygen consumption, and may partly explain these findings (35,36). The relative contributions of each factor may differ from patient to patient, depending on treatment, activity, and the degree and type of sympathetic activation. Variation in autonomic activity may cause changes in coronary endothelial function (36). Alternatively, it may simply modulate the threshold for manifestation of myocardial ischemia in the presence of underlying vascular dysfunction.

## "Occult" Atherosclerosis

Recent pathophysiological studies demonstrate that the current concept of myocardial ischemia induced by epicardial coronary functional or fixed luminal narrowing should be renewed. Acute coronary syndromes often result from disruption of modestly stenotic plaques, not detectable by angiography, but only by intravascular ultrasound (37-39). Plaque rupture and erosion often lead to thrombotic complications (37), or occasionally plaques may rupture and debris may be washed downstream leading to peripheral coronary microembolization often associated with rhythm abnormalities (40-42). Disturbed microvascular integrity could therefore be due to "hidden" epicardial atherosclerosis and its complications.

# Prognosis

Prognosis of patients with chest pain and normal or near normal coronary arteries at angiography is not as benign as reported by preliminary cohort studies (43-46). Many patients are at an increased risk of myocardial infarction and cardiac death. Outcomes of patients (men and women) with unstable angina and non-ST-segment elevation myocardial infarction include 2% of death and myocardial infarction just after 1-year follow-up (47). The risk is not invariably high and the TIMI Risk Score helps to estimate risk. The rate of death or non-fatal myocardial infarction climbs from 0.6% in patients with a TIMI score of 1, to 4.1% in those with a score of 4 or more. Adverse outcomes may also occur in less acute clinical conditions. The combined risk of death, myocardial infarction, stroke, and heart failure is >2% per year in women with a history of chronic chest pain symptoms persisting more than 1 year (48).

**Coronary vascular dysfunction**

The population is heterogeneous and the presence of coronary vascular dysfunction may predict the likelihood of patients to develop coronary events (3).

Coronary vascular dysfunction due to abnormal endothelium-dependent coronary vasodilatation is predictive of adverse outcomes (9,10,49-51). Conversely, impaired endothelial­-independent vasodilatation predicts favorable outcomes (9,49,50). The reason of this discrepancy is not known. One might hypothesize that a blunted response to exogenous nitric oxide donors (coronary endothelial-independent vasodilatation) might simply reflect the presence of atherosclerosis, leading to increased stiffness of the vessel wall [20]. Conversely, endothelial dysfunction and atherosclerosis, although causally related, are distinct problems and may exist separately. Coronary endothelial vasoreactivity may indeed represent an index that combines information of the underlying atherosclerotic process and the overall stress imposed by risk factors on the arterial wall.

**Coronary endothelial dysfunction**

Endothelial dysfunction is an early event in atherosclerosis and has been demonstrated in subjects with risk factors, metabolic syndrome or a family history of premature coronary artery disease (CAD) (52-54).

Coronary endothelial dysfunction in patients with significant CAD provides prognostic value independent of the traditional cardiovascular risk factor assessment (9,10,49-51).

A number of studies addressed the long-term prognostic value of endothelial function testing in patients with non-obstructive (20% to 40% stenosis) CAD. Suwaidi et al. examined 157 patients and reported that there were significantly more cardiovascular events over a 2-year follow-up in patients with endothelial dysfunction (50). Schachinger et al. and von Mering et al. noted similar findings over a 7- and 4-year follow-up, respectively (49,51).

Recent investigations addressed this issue in patients with completely normal coronary angiography and demonstrated that 30% of women with chest pain and severe endothelial dysfunction, as assessed by intra-coronary acetylcholine testing, developed angiographically visible atherosclerosis during a 10-year follow-up (10). Halcox et al. evaluated 176 patients with normal coronary angiography for a mean follow-up of 46 months (9). Endpoints of analysis were cardiovascular death, acute myocardial infarction, unstable angina pectoris, and acute ischemic stroke. Acute vascular events occurred in 4.5% of patients. When patients were divided into 2 groups with either normal or abnormal endothelial function, the event rate of patients with endothelial dysfunction increased up to 14%.

These data extend previous findings showing that peripheral endothelial dysfunction might contribute to the acute ischemic manifestations of CAD. On the one hand, there are patients with diseased coronary arteries and modestly impaired endothelial function. On the other hand there are patients with angiographically normal coronary arteries and severe endothelial dysfunction. Patients with relatively preserved endothelial function have low event rates irrespective of the degree of visible atherosclerosis (56).

# Clinical Decision Making

Nearly 4 million cardiac catheterizations are performed annually in U.S. hospitals alone with 1 in 10 discharges having undergone coronary arteriography (57). More than 20% of patients aged 45-79 years admitted to hospital, receive diagnostic cardiac catheterization (57). More than 50% of women who are referred for cardiac catheterization because of chest pain show non-obstructive coronary lesions or smooth coronary arteries (58). Most of these patients have no diagnosis at the time of discharge. Epidemiological studies demonstrate that undiagnosed angina is costly in terms of mortality, morbidity, and healthcare utilization (59). Sixty-five percent of patients reported angina again during an 11-year follow-up and still remained without a diagnosis. Among those with an abnormal ECG result, the absolute risk of non-fatal myocardial infarction was similar in those with and without a diagnosis (16% versus 15%). Also, compared with apparently healthy subjects, those with undiagnosed and diagnosed angina had a 2.4 and 3.2 times greater risk, respectively, of impaired physical functioning (60). These findings underscore the importance of greater vigilance in identifying the causes of angina in patients with normal angiography.

**Symptoms and Quality of Life**

Chest pain is the most common symptom of coronary atherosclerosis prompting subjects to seek medical attention. Patients may present with stable or unstable symptoms. Interpretation of early symptoms plays a key role in the recognition of patients with normal coronary arteries at angiography, but at risk of future development of atherosclerosis and coronary events (10,48,61,62).

At one extreme are patients in whom angina develops every time the workload of the heart is increased beyond a fairly fixed threshold; at the other extreme are patients who are not necessarily restricted in their physical activity by angina, but suffer rest pain without an obvious cause. Effort that provokes angina on one day may be performed without angina on another day. Both women and men most commonly (80%) report typical angina (10). A number of women report pain as more intense and long lasting (10,48,61,62). They use more emotional words to describe chest pain (10,48). Moreover, their pain is less frequently judged by cardiologists to be typical of angina of cardiac origin (10,48). Chest pain persisting for many years is associated with future development of coronary atherosclerosis (10,48) and adverse outcomes (48).

Along with severe and often unpredictable symptoms, chest pain is often associated with increased psychological morbidity, debilitating symptoms and a poor quality of life (63). Women with chest pain and normal and near normal coronary arteries at angiography have higher levels of anxiety and depression than their CAD counterpart and healthy age-matched subjects. Depression is related to social support and recent traumatic life events (48,64). Patients whose chest pain failed to improve or increased in frequency in the 12 months following angiography had significantly higher psychiatric morbidity at the baseline assessment than patients whose chest pain frequency and severity had abated (65).

*In clinical practice, symptoms in most of these patients are often indistinguishable from those with obstructive CAD. More information and additional testing is needed. It is always important to find whether or not myocardial ischemia exists.*

**Noninvasive Testing**

The majority of patients presenting with chest pain do not undergo invasive investigation immediately, and the cardiologist, following history, physical examination, and imaging or non-imaging exercise stress test, usually makes a diagnosis of the pain as cardiac or non-cardiac.

Non-imaging exercise testing is less sensitive in women than in men (66). The lower prevalence of severe CAD and the fact that more women than men fail to reach maximum aerobic capacity during exercise may account for this difference in sensitivity (66). Moreover, the overall prognostic value of non-imaging exercise testing is under debate. If an exercise test is performed only up to a low level of workload then it may be normal in a proportion of patients who will experience subsequent coronary events (67). Conversely, positive tests showing ST-segment depression may not correspond to any underlying coronary lesion and these "falsely positive" tests are more common in women (68). Mechanisms that may contribute to gender difference remain unclear and may be related to differential effects of estrogens on the ST-segment (69) or differences in vascular reactivity (70).

Use of imaging rather than non-imaging stress testing may be helpful in the identification of microvascular flow obstruction. Magnetic resonance imaging and gated-single photon emission computed tomography (SPECT) show a substantial overlap in detection of adequate or inadequate flow reserve patterns (17,71). In patients with a normal resting ECG, exercise stress myocardial perfusion SPECT yields additional prognostic value over clinical, historical, and exercise treadmill test data for the prediction of coronary events (72). SPECT abnormality is predictive of both cardiac death and myocardial infarction in patients with obstructive and non-obstructive CAD (73,74). Perfusion imaging, unlike angiography, closely correlates with flow reserve and reflects function of the epicardial conduit arteries as well as normal capacity of the resistance vessels (72).

A growing number of centers offer sophisticated cardiac diagnostic tests to aid diagnosis and management of patients with ischemic heart disease. As a consequence, use of magnetic resonance imaging instead of SPECT for initial diagnostic imaging of patients who have chest pain has become more common (17,71). There are exciting new methods that have a potential role in evaluating coronary flow reserve. High frequency transthoracic Doppler harmonic echocardiography appears to give reliable flow measurements (75). Transesophageal echocardiography with Doppler imaging has become a fascinating and appealing tool for diagnosis in routine cardiological practice (76). These new methods deserve additional study to compare imaging time, cost, clinical outcome and patient satisfaction if used as an alternative to conventional SPECT.

*In clinical practice, ischemia with standard SPECT testing identifies symptomatic patients at relatively high risk of a subsequent cardiac event.*

## Routine Coronary Angiography

Slightly more than half of patients who have an imaging stress test subsequently undergo coronary angiography and nearly half of those who had angiography later undergo coronary revascularization (77). These data reflect underlying uncertainties about when to test for CAD. They also demonstrate that identification of non­-obstructive CAD is a frequent clinical finding.

Data analysis of patients undergoing their first coronary angiography over a 3-year period showed that 32% had entirely normal coronary angiograms and an additional 15% had <50% stenosis in any of the major vessels (78). The frequency of non-obstructive CAD is higher in black patients and in women (2,78). Chronic stable angina is the most common clinical presentation in patients without obstructive CAD (78). Many of these patients do not have angiographically visible plaques, but they have coronary disease. The heterogeneity of this patient population may complicate patient selection for appropriate medical management.

*In clinical practice, new effective technologies may provide better understanding of the mechanisms that account for chest pain in patients with normal or near normal angiography*.

## Coronary Flow and Pressure Measurements

Sensor-tipped guidewires have enabled cardiologists to identify physiological measures as absolute coronary flow reserve, relative coronary flow reserve, and pressure-derived fractional flow reserve, the most frequently used being absolute coronary flow reserve (79). Coronary flow reserve is commonly expressed as the ratio between maximal myocardial blood flow after abolition of arteriolar tone and resting baseline flow. Threshold values <2-3 are often associated with diagnosis of microvascular dysfunction (15,74). Coronary flow reserve measurements can however be misleading. A ratio <2-3 can be due either to an increased resting flow or reduced maximal flow, or both. High perfusion pressure due to hypertension is a cause of increased baseline flow and may be a source of significant error (74,79).

*In summary, coronary blood flow measurements by Doppler can be used to identify vascular dysfunction in patients with normal or near normal coronary angiograms. Caution should be used when evaluating patients with hypertension.*

### Intracoronary Ultrasound

In the early stages of atherosclerosis, compensatory enlargement of arterial diameter is an important mechanism for preserving luminal size despite plaque growth (80). The adaptive arterial remodeling response to plaque accumulation is limited and appears to be exhausted when plaque size exceeds a cross-sectional area of 45% or of an increase of 60% of the vessel size circumference (80).

Intravascular ultrasound (IVUS) is a relatively new modality for assessment of atherosclerotic disease burden. IVUS demonstrated that atherosclerotic disease is diffuse and involves the entire arterial tree, including multiple plaques that are not associated with vessel narrowing (37,81-83). Early atherosclerosis, as assessed by IVUS does not necessarily correlate with impairment of coronary blood flow as documented by intracoronary Doppler ultrasound. In a series of 96 patients with normal coronary angiograms more than 50% had signs of early atherosclerosis with IVUS, but only 20% had a reduced coronary flow reserve (83).

*In summary, early signs of atherosclerosis can be detected by IVUS. This may have important therapeutic and prognostic implications (84)*.

## Markers of Early Stage Atherosclerosis and Disease Activity

Numerous studies have examined the relationship between a large number of intermediate markers of cardiovascular disease and the presence of angina with normal angiography. Based on the available data, it could be postulated that most patients with angina and normal angiography have pre-clinical atherosclerosis as defined by raised C-reactive protein, endothelin, and adhesion molecules (85-87). This view is further supported by other observations. For example, C-reactive protein correlates with carotid intima-media thickness and vascular stiffness (88), which, in turn, has been shown to be an independent predictor of adverse cardiovascular events (89,90). It is, therefore, likely that the detection of preclinical atherosclerosis will represent an invaluable tool for future risk stratification of these patients. However, several issues should be addressed before recommending such a strategy in clinical practice. The first one is the choice of the marker to be used for detecting preclinical atherosclerosis. This has not yet been resolved as discussed in a number of different studies (91,92). The second problem is related to the diagnostic value of the marker with regard to the presence of coronary disease and to its predictive value of the occurrence of subsequent coronary events. A typical example is C-reactive protein, it predicts cardiovascular outcome despite the fact that it is a non-specific marker of atherosclerosis. Larger clinical studies are required to determine whether markers of subclinical atherosclerosis may have prognostic significance and whether they should be routinely used.

*To date, it is important to underline that loss of endothelial function coexists and correlates with other cardiovascular markers of early atherosclerosis, such as degree of oxidative stress, plasma levels of C-reactive protein, and intima-media thickness.*

## Endothelial Function Testing

Over the past several years, sophisticated techniques have evolved to meausure endothelium­-dependent vasodilatation in the brachial and coronary arteries.

Peripheral endothelial function testing through measurements of flow-mediated vasodilatation is attractive because it is non-invasive and allows repeated measurements (93).

This technique measures the flow increase through the brachial artery caused by the post­-ischemic dilatation in the downstream vascular bed of the distal forearm. Ischemia is achieved by inflating a cuff placed around the forearm. Measurements are performed by ultrasound. Shear stress and alterations in hydrostatic pressure during reactive hyperemia result in the local release of nitric oxide and activation of endothelium-dependent pathways. The degree of dilatation of the studied vessel from baseline to peak hyperemia reflects the degree of endothelial function. A recent publication has recommended standardization of this methodology (94).

A new, more objective method of measuring 'global' endothelial function has been developed utilizing waveform analysis and beta-2 stimulation of the endothelium using inhaled salbutomol. This technique is fully mobile, uses a laptop computer and applanation tonometry and is ideal for assessing endothelial function in large numbers of subjects (95).

Coronary artery endothelial function is most commonly assessed by intracoronary infusion of acetylcholine, which, acting via muscarinic receptors on endothelial cells, causes release of nitric oxide and coronary artery dilation. Reduced vasodilatory response of the coronary microcirculation or paradoxical vasoconstriction of the epicardial vessels is a sign of coronary endothelial dysfunction (9,10,49-51).

In summary, intracoronary acetylcholine testing may be used for patients who, during angiography, turn out not to have obstructed coronary vessels. Endothelial function measurement by peripheral testing may be a useful surrogate to reduce costs, inconvenience, and risks associated with invasive techniques during medical follow-up.

## Approach to Management of Individual Patients

There are currently no critical pathways specifically developed for patients with chest pain and normal angiograms. So far, physicians apply in varying proportions, pathophysiologic reasoning, personal clinical experience and published research in the development of their own clinical approaches.

Treatment objectives include symptom relief, and risk factor identification and management with the objective of reducing progression in the underlying disease process with enhancement of event-free survival.

## Symptom relief

Few patients respond solely to reassurance. Patients with persistent symptoms are usually regarded as difficult to treat. Perpetuation of symptoms, distress, disability, and continuing concern about heart disease leads to increased rates of re-hospitalization and often to repeat angiography (1,2). Symptoms represent a major burden for health care providers and for patients themselves. Patients complain that their physicians fail to grasp is how life-altering and life-limiting this condition is.

The optimal treatment for patients who have severe symptoms is unclear. Calcium­ antagonists, nitrates and hormone replacement therapy seem to have little effect in preventing chest pain during daily life (32,55,96-100). Beta-adrenergic blockers appear to be highly effective, especially for patients with exertional angina (32,96,101). Imipramine improves the symptoms of some with abnormal cardiac pain perception and “normal” coronary angiograms, possibly through a visceral analgesic effect (102,103). Supplementation of L-arginine, statins and angiotensin-converting enzyme inhibitors may counteract oxidative stress, improve endothelial function, and also reduce symptoms (55,104-106).

*In summary, much more research is needed in this area*. *At present, all patients with chronic, stable ischemic heart disease should receive long-term treatment with beta-blockers to control angina even if they have normal or near normal coronary arteries at angiography. Assuming that the patient has no evidence of myocardial ischemia and/or vascular dysfunction on image stress testing, they can be discharged on imipramine in an attempt to manage chest pain. Patients without evidence of a cardiac etiology may require referral for evaluation of noncardiac causes of chest pain*

**Risk Factor Control and Enhancement of Event-free Survival**

Lifestyle changes, risk factor management and physical training should be considered essential components of any therapeutic approach (107). Diets low in saturated and trans-unsaturated fat and high in fruits, vegetables, fish, and whole grains are associated with better health outcomes (108-110). Physical activity and fitness reduce morbidity and mortality for CHD (111,112).

There is fair or good evidence that aspirin, beta-blockers, statins, and angiotensin-converting enzyme inhibitors, but not nitrates, reduce the risk for cardiovascular events in patients with known heart disease (57). However, there are no data to “prove” that these drugs improve long-term cardiovascular prognosis in patients with chest pain and normal or near normal angiography. Their use for prevention of adverse coronary events means advising that these drugs should be taken on an indefinite basis. Patients are confronted with the possibility that the risk of correcting risk by the indefinite use of some drugs might be greater than the uncorrected risk (113). The question is whether and to what extent aggressive anti-atherosclerotic therapies should be prescribed to these patients? The clinical scenario is pivotal in the choice of therapeutic strategies. An acute coronary event marks a patient for a high long-term risk of recurrence independent of what coronary anatomy look like and thus justifies an aggressive strategy. Clinical guidelines recommend managing all patients with acute coronary syndromes with a standard set of therapies, independent of what coronary anatomy looks like (114). Chronic stable symptoms may require a more selective intervention. Therapy with statins and, perhaps, angiotensin-converting enzyme inhibitors should be used for patients with evidence of coronary endothelial dysfunction and/or atherosclerotic plaques within the arterial wall detected by IVUS (2,82,101). Finally, it would be unethical not to intervene in those patients who qualify for these treatments by the presence of traditional risk factors.

*In summary, a negative angiographic test cannot alleviate the call for intensive lifestyle changes in these patients. The medical community should become increasingly aware that atherosclerosis poses a serious health risk even in its mild form. It is important to understand that the presence of coronary endothelial dysfunction and/or atherosclerotic plaques within the arterial wall is NOT normal and deserves medical intervention.*

# Non-obstructive Coronary Disease in Women

Patients with angina and normal or near normal coronary arteries are predominantly women (115,116). Of interest, the prevalence of non-obstructive disease in women depends on the pattern of angina. It has been noted that one half of women with suspected angina have no obstructive CAD at angiography (58). The prevalence is lower in the setting of more specific clinical symptoms. Post hoc analysis of large clinical trials demonstrates that 10% to 25% of women presenting with acute coronary syndrome have normal or non-obstructive CAD, compared to 6% to 10% in men (47,117-119). This finding raises the possibility that women are being inappropriately referred for coronary angiography; a point contradicted by studies documenting underutilization of cardiac catheterization in women (120). This “female predominance” could be related to hormonal factors. The "estrogen hypothesis" is supported by a number of observations. The Milwaukee Cardiovascular Disease Registry showed a reduced odds ratio for moderate to severe coronary occlusion on angiography in women using estrogen compared with non-users (121). Recently, Husak and colleagues found that unopposed estrogen therapy might have a protective effect on CAD (122). Finally, data from the Women's Ischemia Syndrome Evaluation study suggest that hypoestrogenemia secondary to disrupted ovarian function of hypothalamic origin may accelerate atherosclerosis (123). However, there is no proof that estrogen replacement therapy has protective effect in women who have gone through menopause (124-126).

Unfortunately, there are no large studies or clinical trials that have looked at gender differences in the clinical characteristics, risk factors and outcomes of patients presenting with angina with normal or near normal angiography.

*In summary, the pathophysiology of myocardial ischemia may be very different in women compared to men.*  *Further research is recommended in order to attempt to identify factors in women that result in an increased vulnerability to develop non-obstructive atherosclerotic lesions and vascular dysfunction when compared to men.*

#### Perspectives

Development of large-scale collaborative clinical trials and post hoc analyses are recommended. Large numbers of patients will need to be studied, as relatively low-risk patients will be included. These studies require considerable communication among all parties: researchers, physicians, hospital administrators and policy planners. This can only be realized by close collaboration between important scientific institutions playing a prominent role in research funding and program development.

## Search strategy and selection criteria

We did a computerized and manual search on PubMed to identify studies, with particular focus on original reports published within the past 20 years. Selection criteria included a judgment about the importance of studies and their relevance to the well-informed general practitioner. Keywords used were “angina with normal angiography”, “angina with normal coronary arteries”, “non-obstructive coronary disease” or “chest pain of non-cardiac origin; ” plus “[a]etiology” “pathophysiology”, “diagnosis”, “classification”, “prognosis”, or “therapy ”. There was a restriction on the language of publication: we searched English-language studies only.

**Conflict of interest statement**

We declare that we have no conflict of interest.

#### References

1. Sullivan AK, Holdright DR, Wright CA, et al. Chest pain in women: clinical, investigative, and prognostic features. *BMJ* 1994;308:883–6.
2. Bugiardini R, Bairey Merz CN. Angina with "normal" coronary arteries: a changing philosophy. *JAMA* 2005;293:477-84.
3. Johnson BD, Shaw LJ, Buchthal SD, et al. Prognosis in women with myocardial ischemia in the absence of obstructive coronary disease: results from the National Institutes of Health-National Heart, Lung, and Blood Institute-sponsored Women's Ischemia Syndrome Evaluation (WISE). *Circulation* 2004;109:2993-9.
4. Arbogast R, Bourassa MG. Myocardial function dur­ing atrial pacing in patients with angina pectoris and normal coronary arteriograms: comparison with patients having significant coronary artery disease. *Am J Cardiol* 1973;32:257-63.
5. Cannon RO, Epstein SE. "Microvascular angina" as a cause of chest pain with angiographically normal coro­nary arteries. *Am J Cardiol* 1988;61:1338-43.
6. Temkin LP, Marcus PI. Nonatherosclerotic myocardial ischemia. *J Am Coll Cardiol* 1983;1:1534-5.
7. Hu G, Qiao Q, Tuomilehto J, Balkau B, Borch-Johnsen K, Pyorala K. DECODE Study Group. Prevalence of the metabolic syndrome and its relation to all-cause and cardiovascular mortality in nondiabetic European men and women. *Arch Intern Med* 2004;164:1066-76.
8. Bugiardini R, Pozzati A, Ottani F, Morgagni GL, Puddu P. Vasotonic angina: a spectrum of ischemic syndromes involving functional abnormalities of the epicardial and microvascular coronary circulation. *J Am Coll Cardiol* 1993;22:417-25.
9. Halcox JP, Schenke WH, Zalos G, et al. Prognostic value of coronary vascular endothelial dysfunction. *Circulation* 2002;106:653-8.
10. Bugiardini R, Manfrini O, Pizzi C, Fontana F, Morgagni G. Endothelial function predicts future development of coronary artery disease. A study on women with chest pain and normal angiograms. *Circulation* 2004;109:2518-23.
11. Zeiher AM, Drexler H, Saurbier H, Just H. Endothelium-mediated coronary blood flow modulation in humans: effects of age, atherosclerosis, hypercholesterolemia and hypertension. *J Clin Invest* 1993;92:652–62.
12. Zeiher AM, Drexler H, Wollschläger H, Just H. Endothelial dysfunction of the coronary microvasculature is associated with impaired coronary blood flow regulation in patients with early atherosclerosis. *Circulation* 1991;84:1984–92.
13. Dayanikli F, Grambow D, Muzik O, Mosca L, Rubenfire M, Schwaiger M. Early detection of abnormal coronary flow reserve in asymptomatic men at high risk for coronary artery disease using positron emission tomography. *Circulation* 1994;90:808–17.
14. Camici PG, Marraccini P, Lorenzoni R, et al. Coronary hemodynamics and myocardial metabolism in patients with syndrome X: response to pacing stress. *J Am Coll Cardiol* 1991;17:1461-70.
15. Reis SE, Holubkov R, Conrad Smith AJ et al. Coronary microvascular dysfunction is highly prevalent in women with chest pain in the absence of coronary artery disease: results from the NHLBI WISE study. *Am Heart J* 2001;141:735-41.
16. Hasdai D, Gibbons RJ, Holmes DR, et al. Coronary endothelial dysfunction is associated with myocardial perfusion defects. *Circulation* 1997;96:3390–95.
17. Panting JR, Gatehouse PD, Yang GZ, et al. Abnormal subendocardial perfusion in cardiac syndrome X detected by cardiovascular magnetic resonance imaging. *N Engl J Med* 2002;346:1948-53.
18. Panza JA. Myocardial ischemia and the pains of the heart. *N Engl J Med* 2002;346:1934-5.
19. Crake T, Canepa-Anson R, Shapiro L, Poole-Wilson PA. Continuous recording of coronary sinus oxygen sat­uration during atrial pacing in patients with coronary artery disease or with syndrome X. *Br Heart J* 1988;59:31-8.
20. Picano E, Lattanzi F, Masini M, Distante A, L'Abbate A. Usefulness of high dose dipyridamole-echocardio­graphy test for diagnosis of syndrome X. *Am J Cardiol* 1987;60:508-12.
21. Panza JA, Laurienzo JM, Curiel RV. Investigation of the mechanism of chest pain in patients with angiographically normal coronary arteries using transesophageal dobutamine stress echocardiography. *J Am Coll Cardiol* 1997;29:293–301.
22. Bottcher M, Botker HE, Sonne H, Nielsen TT, Czernin J. Endothelium-dependent and ‑independent perfusion reserve and the effect of L-arginine on myocardial perfusion in patients with syndrome X. *Circulation* 1999;99:1795-801.
23. Buffon A, Rigattieri S, Santini SA, et al. Myocardial ischemia-reperfusion damage after pacing-induced tachycardia in patients with cardiac syndrome X. *Am J Physiol Heart Circ Physiol* 2000;279:H2627–33.
24. Buchthal SD, den Hollander JA, Merz CN, et al. Abnormal myocardial phosphorus-31 nuclear magnetic resonance spectroscopy in women with chest pain but normal coronary angiograms. *N Engl J Med* 2000;342:829-35.
25. Cannon RO III, Dilsizian V, O'Gara PT, et al. Myocardial metabolic, hemodynamic, and electrocardiographic significance of reversible thallium-201 abnormalities in hypertrophic cardiomyopathy. *Circulation* 1991;83:1660-7.
26. Stolen KQ, Kemppainen J, Kalliokoski KK, et al. Myocardial perfusion reserve and peripheral endothelial function in patients with idiopathic dilated cardiomyopathy. *Am J Cardiol* 2004;93:64-8.
27. Strauer BE, Brune I, Schenk H, Knoll D, Perings E. Lupus cardiomyopathy: cardiac mechanics, hemodynamics, and coronary blood flow in uncomplicated systemic lupus erythematosus. *Am Heart J* 1976;92:715-22.
28. Egashira K, Inou T, Hirooka Y, Yamada A, Urabe Y, Takeshita A. Evidence of impaired endothelium-dependent coronary vasodilatation in patients with angina pectoris and normal coronary angiograms. *N Engl J Med* 1993;328:1659-64.
29. Drexler H, Zeiher AM, Wollschlager H, et al. Flow-dependent coronary artery dilatation in humans. *Circulation* 1989;80:466-74.
30. Nabel EG, Ganz P, Gordon JB, Alexander RW, Selwyn AP. Dilation of normal and constriction of atherosclerotic coronary arteries caused by the cold pres­sor test. *Circulation* 1988;77:43-52.
31. Zeiher AM, Krause T, Schachinger V, et al. Impaired endothelium-dependent vasodilation of coronary resistance vessels is associated with exercise-induced myocardial ischemia. *Circulation* 1995;91:2345–52.
32. Bugiardini R, Borghi A, Biagetti L, Puddu P. Comparison of verapamil versus propranolol therapy in syndrome X. *Am J Cardiol* 1989;63:286-90.
33. Lanza GA, Stazi F, Colonna G, Pedrotti P, Manzoli A, Crea F, Maseri A. Circadian variation of ischemic threshold in syndrome X. *Am J Cardiol* 1995;75:683-6.
34. Balady G, Weiner DA, McCabeE CH, Ryan TJ. Value of arm exercise testing in detecting coronary artery disease. *Am J Cardiol* 1985;55:37-39.
35. Montorsi P, Fabbiocchi F, Loaldi A, et al. Coronary adrenergic hyperreac­tivity in patients with syndrome X and abnormal elec­trocardiogram at rest. *Am J Cardiol* 1991;68:1698-1703.
36. Harris KF, Matthews KA. Interactions between autonomic nervous system activity and endothelial function: a model for the development of cardiovascular disease. *Psychosom Med* 2004;66:153-64.
37. Tuzcu EM, Kapadia SR, Tutar E, et al. High prevalence of coronary atherosclerosis in asymptomatic teenagers and young adults: evidence from intravascular ultrasound. *Circulation* 2001;103:2705-10.
38. Jeremias A, Ge J*,* Erbel R. New insight into plaque healing after plaque rupture with subsequent thrombus formation detected by intravascular ultrasound. *Heart.* 1997;77:293.
39. Ge J, Haude M, Görge G, Liu F, Erbel R. Silent healing of spontaneous plaque disruption demonstrated by intravascular ultrasound. *Eur Heart J* 1995;16:1149-51.
40. Skyschally A, Erbel R, Heusch G. Coronary microembolization. *Circ J*  2003;67:279-86.
41. Heusch G, Schulz R. Pathophysiology of coronary microembolisation. *Heart* 2000;89:981‑2.
42. Erbel R, Heusch G. Coronary microembolization. *J Am Coll Cardiol* 2000;36:22-4.
43. Kemp HG, Kronmal RA, Vlietstra RE, Frye RL. Seven years survival of patients with normal or near normal coronary arteriograms: a CASS Registry study. *J Am Coll Cardiol* 1986;7:479-83.
44. Papanicolaou MN, Califf RM, Hlatky MA, et al. Prognostic implications of angiographycally normal and insignificantly narrowed coronary arteries. *Am J Cardiol* 1986;58:1181-7.
45. Lichtlen PR, Bargheer K, Wenzlaff P. Long-term prognosis of patients with anginalike chest pain and normal coronary angiographic findings. *J Am Coll Cardiol* 1995;25:1013-8.
46. Pitts WR, Lange RA, Cigarroa JE, Hillis LD. Repeat coronary angiography in patients with chest pain and previously normal coronary angiogram. *Am J Cardiol* 1997;80:1086-7.
47. Bugiardini R, Manfrini O, De Ferrari GM. Unanswered questions for management of acute coronary syndrome. Risk stratification of patients with minimal disease or normal coronary angiography. *Arch Int Med* 2006;166:1391-5.
48. Johnson BD, Shaw LJ, Pepine CJ, et al. Persistent chest pain predicts cardiovascular events in women without obstructive coronary artery disease: results from the NIH-NHLBI-sponsored Women's Ischaemia Syndrome Evaluation (WISE) study. *Eur Heart J* 2006;27:1408-1.
49. von Mering GO, Arant CB, Wessel TR, et al. Abnormal coronary vasomotion as a prognostic indicator of cardiovascular events in women: results from the National Heart, Lung, and Blood Institute-Sponsored Women's Ischemia Syndrome Evaluation (WISE). *Circulation* 2004;109:722-5.
50. Suwaidi JA, Hamasaki S, Higano ST, Nishimura RA, Holmes DR Jr, Lerman A. Long-term follow-up of patients with mild coronary artery disease and endothelial dysfunction. *Circulation* 2000;101:948-54.
51. Schachinger V, Britten M, Zeiher A. Prognostic impact of coronary vasodilator dysfunction on adverse long-term outcome of coronary heart disease. *Circulation* 2000;101:1899–1906.
52. Reddy KG, Nair RN, Sheehan HM, et al. Evidence that selective endothelial dysfunction may occur in the absence of angiographic or ultrasound atherosclerosis in patients with risk factors for atherosclerosis. *J Am Coll Cardiol* 1994;23:833–43.
53. Schachinger V, Britten MB, Elsner M, et al. A positive family history of premature coronary artery disease is associated with impaired endothelium-dependent coronary blood flow regulation. *Circulation* 1999;100:1502-8.
54. Heitzer T, Schlinzig T, Krohn K, Meinertz T, Munzel T. Endothelial dysfunction, oxidative stress, and risk of cardiovascular events in patients with coronary artery disease. *Circulation* 2001;104:2673-8.
55. Pizzi C, Manfrini O, Fontana F, Bugiardini R. Angiotensin-converting enzyme inhibitors and 3-hydroxy-3-methylglutaryl coenzyme a reductase in cardiac Syndrome X: role of superoxide dismutase activity. *Circulation* 2004;109:53-8.
56. Neunteufl T, Heher S, Katzenschlager R, Wolfl G, Kostner K, Maurer G, Weidinger F. Late prognostic value of flow-mediated dilation in the brachial artery of patients with chest pain. *Am J Cardiol* 2000;86:207-10.
57. Agency for Healthcare Research and Quality, Procedures in U.S. Hospitals, 1997.
58. Sharaf BL, Pepine CJ, Kerensky RA, et al. Detailed angiographic analysis of women with suspected ischemic chest pain (pilot phase data from the NHLBI-sponsored Women's Ischemia Syndrome Evaluation [WISE] Study Angiographic Core Laboratory). *Am J Cardiol* 2001;87:937-41.
59. Hemingway H, Shipley M, Britton A, Page M, Macfarlane P, Marmot M. Prognosis of angina with and without a diagnosis: 11 year follow up in the Whitehall II prospective cohort study. *BMJ* 2003;327:895.
60. Pepine CJ, Balaban RS, Bonow RO, et al. Women's Ischemic Syndrome Evaluation: current status and future research directions: report of the National Heart, Lung and Blood Institute workshop: October 2-4, 2002: Section 1: diagnosis of stable ischemia and ischemic heart disease. *Circulation* 2004;109:e44-6.
61. Maseri A, Crea F, Kaski JC, Crake T. Mechanisms of angina pectoris in syndrome X. *J Am Coll Cardiol* 1991;17:499-506.
62. Bugiardini R. Women, 'non-specific' chest pain, and normal or near-normal coronary angiograms are not synonymous with favourable outcome. *Eur Heart J* 2006;27:1387-9.
63. Asbury EA, Collins P. Psychosocial factors associated with noncardiac chest pain and cardiac syndrome X. *Herz* 2005;30:55-60.
64. Asbury EA, Creed F, Collins P. Distinct psychosocial differences between women with coronary heart disease and cardiac syndrome X. *Eur Heart J* 2004;25:1695-701.
65. Bass C, Wade C, Hand D, Jackson G. Patients with angina with normal and near normal coronary arteries: clinical and psychosocial state 12 months after angiography. *Br Med J* (Clin.Res.Ed) 1983;287:1505-8.
66. Hlatky MA, Pryor DB, Harrel FE Jr, Califf RM, Mark DB, Rosati RA. Factors affecting sensitivity and specificity of exercise electrocardiography. Multivariable analysis. *Am J Med* 1984;77:64-71.
67. Ashley EA, Myers J, Froelicher V. Exercise testing in clinical medicine. *Lancet* 2000;356:1592-7.
68. Coplan NL, Fuster V. Limitations of the exercise test as a screen for acute cardiac events in asymptomatic patients. *Am Heart J* 1990;119:987-90.
69. Bokhari S, Bergmann SR. The effect of estrogen compared to estrogen plus progesterone on the exercise electrocardiogram. *J* *Am Coll Cardiol* 2002;40:1092-6.
70. Palinkas A, Toth E, Amyot R, Rigo F, Venneri L, Picano E. The value of ECG and echocardiography during stress testing for identifying systemic endothelial dysfunction and epicardial artery stenosis. *Eur Heart J* 2002;23:1587-95.
71. Doyle M, Fuisz A, Kortright E, et al. The impact of myocardial flow reserve on the detection of coronary artery disease by perfusion imaging methods: an NHLBI WISE study. *J Cardiovasc Magn Reson* 2003;5:475-85.
72. Hachamovitch R, Berman DS, Kiat H, Cohen I, Friedman JD, Shaw LJ. Value of stress myocardial perfusion single photon emission computed tomography in patients with normal resting electrocardiograms: an evaluation of incremental prognostic value and cost-effectiveness. *Circulation* 2002;105:823-9.
73. Abdel Fattah A, Kamal AM, Pancholy S, et al. Prognostic implications of normal exercise tomographic thallium images in patients with angiographic evidence of significant coronary artery disease. *Am J Cardiol* 1994;74:769–71.
74. Wieneke H, Zander C, Eising EG, Haude M, Bockisch A, Erbel R. Non-invasive characterization of cardiac microvascular disease by nuclear medicine using single-photon emission tomography. *Herz* 1999;24:515-21.
75. Bartel T, Yang Y, Muller S, et al. Noninvasive assessment of microvascular function in arterial hypertension by transthoracic Doppler harmonic echocardiography. *J Am Coll Cardiol* 2002;39:2012-8.
76. Poelaert JI, Schupfer G. Hemodynamic monitoring utilizing transesophageal echocardiography: the relationships among pressure, flow, and function. *Chest* 2005;127:379-90.
77. Wennberg DE, Kellett MA, Dickens JD, Malenka DJ, Keilson LM, Keller RB. The association between local diagnostic testing intensity and invasive cardiac procedures. *JAMA* 1996;275:1161-4.
78. Mathew J, Krishna A, Hallak AA, et al Clinical and angiographic findings in black patients with suspected coronary artery disease. *Int J Cardiol* 1997;62:251-7.
79. Strauer BE. The significance of coronary reserve in clinical heart disease. *J Am Coll Cardiol* 1990;15:775-83.
80. Glagov S, Weisenberg E, Zarins CK, Stankunavicius R, Kolettis GJ. Compensatory enlargement of human atherosclerotic coronary arteries. *N Engl J Med* 1987;316:1371-5.
81. Erbel R, Ge J, Görge G, et al. Intravascular ultrasound classification of atherosclerotic lesions according to American Heart Association recommendation. *Coron Artery Dis* 1999;10:489-99.
82. Nissen SE, Tuzcu EM, Schoenhagen P, et al. Effect of intensive compared with moderate lipid-lowering therapy on progression of coronary atherosclerosis: a randomized controlled trial. *JAMA* 2004;291:1071-80.
83. Erbel R, Ge J, Bockisch A, et al.. Value of intracoronary ultrasound and Doppler in the differentiation of angiographically normal coronary arteries: a prospective study in patients with angina pectoris. *Eur Heart J* 1996;17:880-9.
84. Smith SC Jr, Feldman TE, Hirshfeld JW Jr, et al. ACC/AHA/SCAI 2005 guideline update for percutaneous coronary intervention: a report of the American College of Cardiology/American Heart Association Task Force on Practice Guidelines (ACC/AHA/SCAI Writing Committee to Update 2001 Guidelines for Percutaneous Coronary Intervention) *Circulation* 2006;113:e166-286.
85. Cosin-Sales J, Pizzi C, Brown S, Kaski JC. C-reactive protein, clinical presentation, and ischemic activity in patients with chest pain and normal coronary angiograms.  *J Am Coll Cardiol* 2003;41:1468-74.
86. Kaski JC, Elliott PM, Salomone O, et al. Concentration of circulating plasma endothelin in patients with angina and normal coronary angiograms. *Br Heart J* 1995;74:620-4.
87. Tousoulis D, Davies GJ, Asimakopoulos G, et al. Vascular cell adhesion molecule-1 and intercellular adhesion molecule-1 serum level in patients with chest pain and normal coronary arteries (syndrome X). *Clin Cardiol* 2001;24:301-4.
88. Arroyo-Espliguero R, Mollichelli N, Avanzas P, et al. Chronic inflammation and increased arterial stiffness in patients with cardiac syndrome X. *Eur Heart J* 2003;24:2006-11.
89. Chan SY, Mancini GB, Kuramoto L, Schulzer M, Frohlich J, Ignaszewski A. The prognostic importance of endothelial dysfunction and carotid atheroma burden in patients with coronary artery disease*. J Am Coll Cardiol* 2003;42:1037-43.
90. Grey E, Bratteli C, Glasser SP, et al. Reduced small artery but not large artery elasticity is an independent risk marker for cardiovascular events. *Am J Hypertens* 2003;16:265-9.
91. Libby P, Ridker PM. Novel inflammatory markers of coronary risk: theory versus practice. *Circulation* 1999;**100**:1148-50.
92. Ashfaq S, Abramson JL, Jones DP, et al. The relationship between plasma levels of oxidized and reduced thiols and early atherosclerosis in healthy adults*. J Am Coll Cardiol* 2006;47:1005-11.
93. Kuvin JT, Karas RH. Clinical utility of endothelial function testing: ready for prime time? *Circulation* 2003;107:3243-7.
94. Corretti, MC, Anderson TJ, Benjamin EJ, et al. Guidelines for the ultrasound assessment of endothelial-dependent flow-mediated vasodilation of the brachial artery: A report of the International Brachial Artery Reactivity Task Force. *J Am Coll Cardiol* 2002;39:257-65.
95. Hayward CS, Kraidly M, Webb CM, Collins P. Assessment of endothelial function using peripheral waveform analysis. A clinical application. *J Am Coll Cardiol* 2002;40:521-8.
96. Lanza GA, Colonna G, Pasceri V, Maseri A. Atenolol versus amlodipine versus isosorbide-5-mononitrate on anginal symptoms in syndrome X. *Am J Cardiol* 1999;84:854-6.
97. Masumoto A, Mohri M, Takeshita A. Three-year follow-up of the Japanese patients with microvascular angina attributable to coronary microvascular spasm. *Int J Cardiol* 2001;81:151-6.
98. Adamson DL, Webb CM, Collins P. Esterified estrogens combined with methyltestosterone improve emotional well-being in postmenopausal women with chest pain and normal coronary angiograms. *Menopause* 2001;8:233-8.
99. Bugiardini R, Borghi A, Pozzati A, Ottani F, Morgagni GL, Puddu P. The paradox of nitrates in patients with angina pectoris and angiographically normal coronary arteries. *Am J Cardiol* 1993;72:343-7.
100. Sutsch G, Oechslin E, Mayer I, Hess OM. Effect of diltiazem on coronary flow reserve in patients with microvascular angina. *Int J Cardiol* 1995;52:135-43.
101. Fox K, Garcia MA, Ardissino D, et al. Guidelines on the management of stable angina pectoris: executive summary: The Task Force on the Management of Stable Angina Pectoris of the European Society of Cardiology. *Eur Heart J* 2006;27:1341-81.
102. Cannon RO III, Quyyumi AA, Schenke WH, et al. Abnormal cardiac sensitivity in patients with chest pain and normal coronary arteries. *J Am Coll Cardiol* 1990;16:1359-66.
103. Cannon RO III, Quyyumi AA, Mincemoyer R, et al. Imipramine in patients with chest pain despite normal coronary angiograms. *N Engl J Med* 1994;330:1411–7.
104. Lerman A, Burnett JC Jr, Higano ST, McKinley LJ, Holmes DR Jr. Long-term L-arginine supplementation improves small-vessel coronary endothelial function in humans. Circulation 1998;97:2123-8.
105. Chen JW, Hsu NW, Wu TC, Lin SJ, Chang MS. Long-term angiotensin-converting enzyme inhibition reduces plasma asymmetric dimethylarginine and improves endothelial nitric oxide bioavailability and coronary microvascular function in patients with syndrome X. *Am J Cardiol* 2002;90:974-82.
106. Kayikcioglu M, Payzin S, Yavuzgil O, Kultursay H, Can LH, Soydan I. Benefits of statin treatment in cardiac syndrome-X1. *Eur Heart J* 2003;24:1999-2005.
107. Mosca Li, Appel LJ, Benjamin EJ, et al. **Evidence-based guidelines for cardiovascular disease prevention in women** *Circulation* 2004;109:672-93.
108. Pietinen P, Rimm EB, Korhonen P, et al. Intake of dietary fiber and risk of coronary heart disease in a cohort of Finnish men: the alpha-tocopherol, beta-carotene cancer prevention study. *Circulation.* 1996;94:2720-7.
109. Marckmann P, Gronbaek M. Fish consumption and coronary heart disease mortality. A systematic review of prospective cohort studies. *Eur J Clin Nutr* 1999;53:585-90.
110. Joshipura KJ, Hu FB, Manson JE, et al. The effect of fruit and vegetable intake on risk for coronary heart disease. *Ann Intern Med* 2001;134:1106-14.
111. Blair SN 3rd, Kohl HW, Barlow CE, Paffenbarger RS Jr, Gibbons LW, Macera CA. Changes in physical fitness and all-cause mortality. A prospective study of healthy and unhealthy men. *JAMA* 1995;273:1093-8.
112. Wessel TR, Arant CB, Olson MB, et al. Relationship of physical fitness vs body mass index with coronary artery disease and cardiovascular events in women *JAMA* 2004;292:1179-87.
113. Oliver MF. Risks of correcting the risks of coronary disease and stroke with drugs. *N Engl J Med* 1982;306:297-8.
114. Braunwald E, Antman EM, Beasley JW, et al. American College of Cardiology; American Heart Association. Committee on the Management of Patients With Unstable Angina. ACC/AHA 2002 guideline update for the management of patients with unstable angina and non-ST-segment elevation myocardial infarction--summary article: a report of the American College of Cardiology/American Heart Association task force on practice guidelines (Committee on the Management of Patients With Unstable Angina) *J Am Coll Cardiol* 2002;40:1366-74.
115. Castelli WP. Epidemiology of coronary heart disease: The Framingham Study. *Am J Med* 1984;76:4-12.
116. Weiner DA, Ryan TJ, McCabe CH, et al: Exercise stress testing: correlations among history of angina, ST-segment response and prevalence of coronary-artery disease in the Coronary Artery Surgery Study (CASS). *N Engl J Med* 1979;301:230-5.
117. Hochman JS, McCabe CH, Stone PH, et al. Outcome and profile of women and men presenting with acute coronary syndromes: a report from TIMI IIIB. *J Am Coll Cardiol* 1997;30:141-8.
118. Hochman JS, Tamis JE, Thompson TD, et al. Sex, clinical presentation, and outcome in patients with acute coronary syndromes. Global Use of Strategies to Open Occluded Coronary Arteries in Acute Coronary Syndromes IIb Investigators. *N Engl J Med* 1999;341:226-32.
119. Anand SS, Xie CC, Mehta S, et al. Differences in the management and prognosis of women and men who suffer from acute coronary syndromes. *J Am Coll Cardiol* 2005;46:1845-51.
120. Laskey WK. Gender differences in the management of coronary artery disease. Bias or good clinical judgment? *Ann Intern Med* 1992;116:869-71.
121. Gruchow HW, Anderson AJ, Barboriak JJ, et al. Postmenopausal use of estrogen and occlusion of coronary arteries. *Am Heart J* 1988;115:954-63.
122. Husak L, Vaccarino V, Veledar E, Murrah N, Wenger NK. Comparison of angiographic findings among postmenopausal women using unopposed estrogen, estrogen/progestin combinations, and nonusers *Am J Cardiol* 2004;93:563-8.
123. Bairey Merz CN, Johnson BD, Sharaf BL, et al. Hypoestrogenemia of hypothalamic origin and coronary artery disease in premenopausal women: a report from the NHLBI-sponsored WISE study. *J Am Coll Cardiol* 2003;41:413-9.
124. Hulley S. Estrogens should not be initiated for the secondary prevention of coronary artery disease: a debate. *Can J Cardiol* 2000;16 Suppl E:10E-12E.
125. Grady D, Brown JS, Vittinghoff E, Applegate W, Varner E, Snyder T; HERS Research Group. Postmenopausal hormones and incontinence: the Heart and Estrogen/Progestin Replacement Study. *Obstet Gynecol* 2001;97:116-20.
126. Rossouw JE, Anderson GL, Prentice RL, et al. Risks and benefits of estrogen plus progestin in healthy postmenopausal women: principal results From the Women's Health Initiative randomized controlled trial. *JAMA* 2002;288:321-33.
